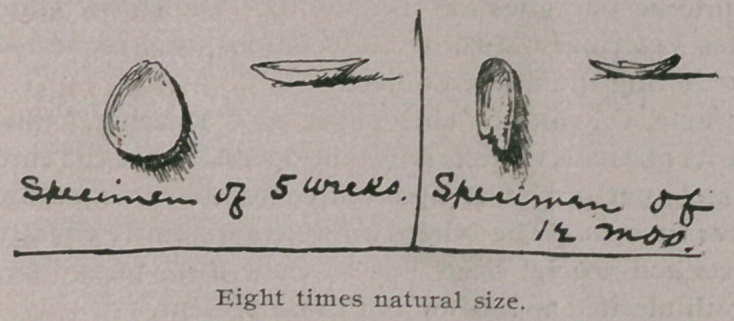# Foreign Bodies Removed from the Eye

**Published:** 1890-03

**Authors:** 


### FOREIGN BODIES REMOVED FROM THE EYE.

Dr. Samuel Theobald exhibited under a low-power microscope the following foreign bodies removed from the eye. They were all, with perhaps one exception, of a similar nature, being parts of the capsules of minute seeds. The exception appeared to be a portion of the wing-covering of a small insect. They were all concave upon one surface and convex upon the other, and each one had been removed from the surface of the cornea, to which it was attached by its concave side. The points of interest were that the peculiar shape of these bodies had caused them to take an exceptionally firm hold upon the cornea, while their structure enabled them to resist for long periods the solvent action of the fluids of the eye. Being, moreover, semi-transparent, their true nature had not in every instance been easily recognized. Of the eight foreign bodies of this character which were exhibited, three had remained attached to the cornea for periods less than two weeks, two for five weeks, one for two months, one for three months, and one for a whole year. Ulceration of the cornea beneath the foreign body had occurred in almost every instance, and in three of the cases small bloodvessels had developed upon the cornea running from the conjunctiva to the point of attachment of the foreign body. Several of the eyes had previously been treated without the presence of a foreign body having been suspected, and in one case "caustic" applications were said to have been made. After the removal of the foreign bodies the eyes all recovered quickly under simple treatment.—*Proceedings Johns Hopkins Hospital Medical Society.*

## Figures and Tables

**Figure f1:**